# Transdiagnostic mental health symptom dimensions predict use of flexible model-based inference in complex environments

**DOI:** 10.1038/s41398-026-03922-w

**Published:** 2026-03-07

**Authors:** Toby Wise, Sirichat Sookud, Giorgia Michelini, Dean Mobbs

**Affiliations:** 1 Department of Neuroimaging, Institute of Psychiatry, Psychology & Neuroscience, King’s College London, London, UK; 2 Centre for Brain and Behaviour, Department of Psychology, School of Biological and Behavioural Sciences, Queen Mary University of London, London, UK; 3 Department of Humanities and Social Sciences and California Institute of Technology, Pasadena, California USA; 4 Computation and Neural Systems Program at the California Institute of Technology, California, USA

**Keywords:** Human behaviour, Psychiatric disorders

## Abstract

Symptoms of common mental health problems often pertain to complex inference and decision problems (for example around future social interactions). Such patterns may reflect the breakdown of the fundamental computational processes that ordinarily underpin these behaviours, with the use of flexible goal-directed decision-making being a prime candidate. Here, we used a validated, naturalistic threat inference task to assess the use of goal-directed decision-making in complex interactive decision problems. Participants (n = 1025) completed this task alongside a battery of self-report measures of mental health symptoms and neurodevelopmental characteristics. Participants higher in inattentive/neurodevelopmental symptoms were better able to predict the predator’s behaviour, while those higher in externalising symptoms made more incorrect inferences. Variability in behaviour was better explained by these specific symptom dimensions than by more general factors. Using computational modelling, we show that these associations are mediated by the degree to which individuals use goal-directed decision-making to make inferences about the predator’s behaviour. Our results suggest that symptoms and traits that manifest in real-world environments may result from alterations in the use of complex computational mechanisms.

## Introduction

Humans are adept at predicting others’ actions and adapting their behaviour accordingly [1]. Such processes are complex and likely depend upon task-general mechanisms that permit flexible, goal-directed decision-making [2, 3]. Our remarkable ability to navigate interactive environments, however, is a breaking point in many mental health problems and neurodevelopmental conditions, with social inference behaviours often linked tosymptoms [4, 5]. Such challenges span traditional psychiatric diagnostic categories, ranging from social anxiety [6] to psychosis [7], but also characterise neurodevelopmental conditions such as attention deficit hyperactivity disorder (ADHD) [8] and autism [9]. It remains an open question to what extent these difficulties reflect a general alteration in flexible, goal-directed decision-making processes that support inferences about others.

A prevalent theme within prior research into the role of decision-making within computational psychiatry is the importance of internal world models [10], which allow for flexible, prospective, and goal-directed planning. The use of this general “model-based” decision-making strategy has been linked across multiple studies to transdiagnostic symptom dimensions [11–15]. Further work has identified links between anxiety and model-based planning when seeking protection from danger specifically [16], suggesting that task context may influence the nature of such symptom-behaviour relationships.

However, it is unclear to what extent model-based decision-making in interactive environments specifically plays a role in the genesis of symptoms. Instead, these processes are typically assessed using behavioural tasks with very simple task structures and no requirement to consider the actions of others. In recent years, efforts have been made to incorporate elements of naturalism into the study of human decision-making [17], and developments in task design and computational modelling have allowed elements of naturalistic complexity, including multi-step social interactions [2, 18, 19], to be embedded within behavioural tasks. This approach has the potential to clarify the role of interactive decision-making processes in mental health and neurodevelopmental conditions.

Moreover, it is important to consider how symptoms themselves are assessed. Research within computational psychiatry has frequently exploited a transdiagnostic approach, identifying mechanisms that underpin largely independent symptom dimensions across diagnostic categories [20]. However, an emerging view, exemplified by the Hierarchical Taxonomy of Psychopathology (HiTOP) system [21, 22], argues that mental health should be treated as a hierarchy of related symptom dimensions (for example, anxiety and mood symptoms nested within internalising symptoms), accounting for the presence of similarities across dimensions. Importantly, neurodevelopmental conditions, such as ADHD and autism, can be integrated into this framework, accounting for their comorbidity and overlap with mental health problems [23].

Here, we explore the role of interactive model-based decision-making in transdiagnostic mental health and neurodevelopmental symptoms, using a more naturalistic approach that better approximates the complexity of real-world social decision-making. We exploit a recently-developed predator-prey task [2] in which participants must infer a virtual predator’s behaviour and predict its goal-directed actions. This enables us to assess how participants used a model of the task to predict another agent’s behaviour. Importantly, this task was not designed to test avoidance *per* se, but instead focuses on “pre-encounter” threat, where participants are in an environment where danger is possible but not imminent, and must predict the predator’s behaviour to increase the likelihood of staying safe. We link behaviour in this task to hierarchically structured mental health and neurodevelopmental dimensions, enabling us to determine the specificity of such relationships to fine-grained symptom dimensions versus broader dimensions. This was intended as an exploratory study; given the paucity of existing literature in this area, we aimed to generate, rather than test, hypotheses.

## Materials and methods

### Sample

Participants were recruited through Prolific [24], and were selected based on having a 95% approval rate and being aged 18 or over and living in either the United Kingdom or United States.

### Task

Participants completed a variant of the task described in our previous work [2]. In summary, the task involved navigating a character (a robot) around a 21×10 2D hexagonal grid environment while avoiding being caught by a predator (represented as a “blob monster”). The task was created using Svelte (https://svelte.dev/) and presented in the web browser. Participants were told that each gold coin they collected was worth 100 points, and 1000 points was worth a £0.20 bonus payment at the end of the task. Getting caught by the predator (i.e., occupying the same cell on the grid as the predator) would end the game, and all the points accumulated in that game would be lost. Participants were told that their goal was to collect as many coins as possible while avoiding getting caught by the predator.

Participants played 4 games, within which they made 10 moves (1 per turn) while the predator made 20 (2 per turn). Each game was played in a different environment, but participants were told that the predator was consistent across games. Participants were not informed of the predator’s preference and instead were asked to report their estimate of its preference for each of the three features (trees, red ground, robot) using sliding scales at the end of each game, alongside reporting their confidence in these ratings. We also asked participants to predict where they thought the predator would move before each of its turns, and they were also asked to rate their confidence in their predictions. Correct predictions influenced the bonus payment, with 5 predictions being selected at random at the end of the task and an additional £0.20 paid for each correct prediction. They were also informed that their confidence ratings would influence the calculation of their bonus payment, with predictions in which they were more confident being more likely to be selected for the bonus payment.

The predator was programmed to behave according to a given set of preferences, seeking out the feature it preferred (here, this preference was always for the trees). This meant that it would not actively “chase” the participant, and they would only be caught if they strayed into its path. In general, the predator was designed to be straightforward to avoid as long as participants correctly inferred its goal, as this enabled the participant to take a path that would easily avoid the predator. A full description of the methods used to determine the predator’s actions is provided in our prior work [2], but in brief, the predator selected actions according to their value as estimated using value iteration. One difference from our prior work is that here we introduced a degree of noise in the predator’s action selection, achieved by setting the temperature parameter of the softmax rule used to choose actions to a value of 0.5. This was done to increase the difficulty of the task, making it more challenging for participants to infer the predator’s preferences and plan their own actions [25].

### Questionnaire measures

#### Symptom measures

We administered a battery of measures assessing various aspects of mental health problems and neurodevelopmental conditions. For consistency, we selected measures targeting trait-level characteristics of these conditions. Specifically, we used the Autism Spectrum Quotient (AQ10 [26]), ADHD Self-Report Scale (ASRS [26]), Community Assessment of Psychic Experiences (CAPE [27]), Externalising Symptom Inventory Short Form (ESI-SF [28]), Personality Inventory for DSM-5 (PID-5 [29]), and State Trait Inventory of Cognitive and Somatic Anxiety Trait scale (STICSA-T [30]). Any reverse coded items were reversed prior to further analysis. This battery included a range of measures designed to target specific symptom domains (AQ10: autism; ASRS: ADHD; CAPE: psychosis; ESI: externalising symptoms; STICSA: anxiety). The PID-5 was also included as it provides items assessing various affective symptoms alongside characteristics associated with personality disorders.

#### Attention checks

We also included a number of items designed to ensure that participants were paying attention to the questions. These included both explicit attention checks (e.g., “Please select strongly agree in response to this question”) and infrequency items [31] (e.g., “I have single-handedly sailed around the world – agree/disagree”). For the explicit attention checks, any response other than the one requested was treated as a failure. For the infrequency items, we treated the 1^st^ and 2^nd^ most “correct” responses as correct and any others were classed as failures.

### Statistical analysis

#### Exclusions

In order to minimise the influence of inattentive responding, we excluded participants who failed any attention checks or more than one infrequency item. We also excluded participants for whom we did not have a full dataset, whether due to failure to complete or due to technical failure. Finally, for analyses including gender as a covariate, we excluded any participants reporting gender other than male or female as there were insufficient numbers to estimate effects related to other categories.

#### Exploratory factor analysis

Exploratory factor analysis (EFA) was performed using Oblimin rotation to allow for correlation between factors. This was conducted using the Factor Analyzer package for Python (https://factor-analyzer.readthedocs.io). The number of factors to retain was determined based on visual inspection of the scree plot and interpretability of resulting factors. We chose this over parallel analysis as we found that parallel analysis tended to produce solutions with very high numbers of factors, the majority of which were not interpretable. This is likely due to our sample size, since large samples result in null eigenvalues close to 1 [32]. Having determined the optimal number of factors, we repeated the process using fewer factors to provide dimensions with decreasing levels of granularity (for example, starting at 4 factors, then repeating this process down to 1 factor in steps of 1). Scores on each of the factors were extracted for each subject, and these were used as variables of interest in further analyses. We also used these scores to determine the hierarchical structure of our factors, calculating correlations between factors at adjacent levels of the hierarchy. Correlations approaching 1 suggested that a factor was retained when moving to a lower level of the hierarchy, while high correlations (between 0.5 and 0.9) indicated that the factor was not present in the higher level but displayed some relation to a factor that was present (i.e., could be viewed as related hierarchically to this higher-level factor).

#### Sequential regression analysis

To determine whether behavioural characteristics were related to the transdiagnostic dimensions, we performed sequential regression analyses [33, 34]. These models were run using the statsmodels package for Python (https://www.statsmodels.org). In each model, we included age, gender, and self-reported motivation for the task as covariates. For models of confidence in movement predictions, we additionally included the proportion of correct responses as a covariate to control for effects of accuracy. For models of confidence in preference ratings, we included absolute error (again to control for accuracy effects), alongside the average rating and the average rating squared as we observed a correlation between confidence and rating extremity (whereby more extreme ratings attracted higher confidence ratings).

For analyses of computational model parameters, we transformed the weighting parameter $W$ using a cubic transform due to the values being heavily skewed. All continuous variables were subsequently scaled to have zero mean and unit variance.

The sequential regression procedure began by predicting the behavioural measure of interest from covariates alone (information on covariates is provided below). The simplest models therefore took the form:

1 $${\rm{y}}={\rm{\beta }}0+{\rm{\beta }}1\times {\rm{Age}}+{\rm{\beta }}2\times {\rm{Gender}}+{\rm{\epsilon }}$$

We then added in the dimensions derived through factor analysis one level at a time, so the next model became:

2 $${\rm{y}}={\rm{\beta }}0+{\rm{\beta }}1\times {\rm{Age}}+{\rm{\beta }}2\times {\rm{Gender}}+{\rm{\beta }}3\times {\rm{General}}+{\rm{\epsilon }}$$

And these models increased in complexity up to the full model with all factors included:

3 $${\rm{y}}={\rm{\beta }}0+{\rm{\beta }}1\times {\rm{Age}}+{\rm{\beta }}2\times {\rm{Gender}}+{\rm{\beta }}3\times {\rm{Ext}}+{\rm{\beta }}4\times {\rm{Int}}+{\rm{\beta }}5\times {\rm{Withdr}}+{\rm{\beta }}6\times {\rm{Inatt}}+{\rm{\epsilon }}$$

We compared the relative model fit of each iteration using a combination of the adjusted ${R}^{2}$ of the models and an ANOVA comparing models sequentially. A significant ANOVA result combined with an increase in adjusted ${R}^{2}$ was taken as evidence that the more granular model explained a significantly greater proportion of the variance in behaviour than the preceding model. Using the adjusted ${R}^{2}$ ensured that increasing model complexity was accounted for. We estimated confidence intervals and significance for individual coefficients using bootstrapping with 20,000 samples. To provide an index of the robustness and generalisability of these models, we also performed 10-fold cross-validation to determine the extent to which our results may be replicated in data that was not used to estimate the model, using ${R}^{2}$ as an index of how well the model captures the held out data. We confirmed the significance of these results using permutation testing, for which we repeated the cross-validation process on data where the target variable was permuted, providing an empirical null distribution against which to compare the true cross-validated estimate.

#### Mediation analysis

To further understand the nature of relationships between behaviour and symptoms, we performed mediation analyses [35] using the pinguoin Python package (https://pingouin-stats.org). This enabled us to establish whether observed associations between symptom dimensions and behavioural patterns were mediated by parameters from our computational models, providing further evidence for the mechanistic role of the processes described by these models. While mediation analysis cannot provide conclusive evidence for a causal model in which symptom–behaviour relationships are mediated by the processes described by these models, they do provide one test of such a model in the absence of more direct experimental tests.

To achieve this, we performed four regression analyses: 1) ${model\; parameter} \sim {symptom}$, 2) ${behaviour} \sim {model\; parameter}$, 3) ${behaviour} \sim {symptom}$, 4) ${behaviour} \sim {symptom}$ (controlling for ${model\; parameter}$). Significant effects for paths 1–3, in conjunction with a significant decrease in the strength of path 4 relative to path 3 provides evidence for a mediation effect. We also estimate the indirect effect (also referred to as the average causal effect of mediation [36]) as an estimate of the strength of mediation. Significance was estimated using bootstrapping with 20,000 samples.

### Computational modelling

The computational modelling approach was near identical to that described in our prior work [2]. These models were designed to capture putative mechanisms underpinning participants’ ability to predict the predator’s upcoming moves and could be either model-free (i.e., not using a model of the task environment) or model-based (i.e., using a model of the task environment).

The simplest of the model-free models is referred to as “policy learning”, and assumes that participants learn the predator’s policy using error-driven learning. Essentially, this model learns the extent to which the predator tends to select a particular action, with no regard to the environment in which it is choosing these actions. Formally, this model learns the predator’s preferred actions as follows:

4 $${\hat{{\rm{\pi }}}}_{t}\left(a\right)={\hat{{\rm{\pi }}}}_{t-1}\left(a\right)+{{\rm{\alpha }}}_{t}\cdot \left({a}_{{t;obs}}-{\hat{{\rm{\pi }}}}_{t-1}\left(a\right)\right)$$

Here, ${\hat{{\rm{\pi }}}}_{t}(a)$ is the estimate of the probability of performing action $a$ after observing the action on trial $t$, ${a}_{{t;obs}}$ is the action observed on that trial and ${{\rm{\alpha }}}_{t}$ is a learning rate parameter. Note that in contrast to our prior work using this model [2], the learning rate did not decay.

We also included an extension of this model that accounted for correlations between adjacent action values, assuming that adjacent action values should be similar. In this model, we convolved the observed action (a one-hot vector representing the one chosen action on the current trial) with a squared exponential kernel:

5 $$k\left(x,{x}_{{\rm{\top }}}\right)=\exp \left[-\frac{{\left(x-{x}_{\mathrm{T}}\right)}^{2}}{2{{\mathscr{L}}}^{2}}\right]$$

As a result, the chosen action was generalised to adjacent actions, resulting in them having similar values, through convolution using this kernel:

$${\hat{{\boldsymbol{a}}}}_{{t;obs}}=k({{\boldsymbol{a}}}_{{t;obs}},{{\boldsymbol{a}}}_{{t;obs}})$$

Where ${{\boldsymbol{a}}}_{{t;obs}}$ is a one-hot vector representing the action chosen by the agent on trial $t$. The estimate of $\hat{{\rm{\pi }}}$ was updated as in Eq. 4, using ${\hat{{\boldsymbol{a}}}}_{{t;obs}}$ in place of ${a}_{{t;obs}}$. The length scale parameter ${\mathscr{L}}$ was fixed at 0.02.

The final model-free model was the simplest, assuming that the predator would simply repeat its previous move. This was achieved by setting the learning rate in Equation 4 to 1.

Our model-based approach was more complex, and used value iteration to determine the optimal policy given a model of the task based on the true preferences of the predator. We additionally fit combinations of the model-free and model-based models, scaling the action values $\hat{{\rm{\pi }}}$ estimated by each model to the range 0–1 to ease interpretation of resulting weights on the values from each model:

6 $$\hat{{\rm{\pi }}}=\frac{\hat{{\rm{\pi }}}-\min \left(\hat{{\rm{\pi }}}\right)}{\max \left(\hat{{\rm{\pi }}}\right)-\min \left(\hat{{\rm{\pi }}}\right)}$$

These action value estimates were then combined according to weighting parameter $W$ (which could take values from 0 to 1).

7 $${\hat{{\rm{\pi }}}}_{{combined}}\left(a\right)=W\cdot {\hat{{\rm{\pi }}}}_{{goal}}\left(s,a\right)+\left(1-W\right)\cdot {\hat{{\rm{\pi }}}}_{{policy}}\left(a\right)$$

We formed 3 combination models by combining the model-based strategy with each of the 3 model-free models. The output of each model was transformed using a softmax function to produce choice probabilities, with the temperature parameter set to 1. We then determined model fit using the log likelihood of the model based on the categorical likelihood function.

8 $$-L\left({\rm{\theta }}\right)=-\mathop{\sum }\limits_{t=1}^{N}\log \left({\hat{{\rm{\pi }}}}_{{combined}}\left({a}_{{t;obs}}\right)\right)$$

Here, ${\hat{{\rm{\pi }}}}_{{combined}}\left({a}_{{t;obs}}\right)$ is the inferred probability of the predator selecting this action on trial $t$, summed across all predictions $N$. Parameters $\theta$ were the learning rate$\,\alpha$ in the policy learning models and the weighting parameter $W$ in the combined models. These were estimated using differential evolution in SciPy (https://scipy.org/). For the purposes of model comparison, we calculated the Bayesian Information Criterion (BIC) of each model. While we focus on the weighting parameter $W$ as our primary measure of the extent to which participants deploy model-based strategies over model-free, we additionally calculate the difference in model fit between models describing pure model-based and model-free strategies (ΔBIC) as an additional measure to provide confirmatory support for our results. All modelling was performed in Python. Alongside analyses of raw behavior, our models provide a number of metrics used in further analyses (Table 1).

**Table 1:** Description of variables examined in regression analyses.

Variable	Source	Definition
Prediction accuracy	Raw behaviour	The accuracy of participants’ predictions of where the predator will move on its next turn (i.e., the percentage of correct predictions)
Prediction confidence	Raw behaviour	Subjective confidence in predictions about the predator’s movements
Rating accuracy	Raw behaviour	The accuracy of participants ratings of the predator’s preference (i.e., the error in their ratings relative to the true preference, which was always for the trees)
Rating confidence	Raw behaviour	Subjective confidence in ratings of the predator’s preferences
*W*	Computational model	Model parameter determining the relative contribution of model-based versus model-free strategies to predictions of the predator’s behaviour. Higher values indicate a greater model-based contribution (using planning to determine the likely behaviour of the predator according to its goals), while lower values indicate a greater model-free contribution (assuming the predator will simply repeat actions it has taken recently).
ΔBIC	Computational model	An additional measure of participants’ tendency to use model-based strategies, representing the difference in model fit between a pure model-based account and a pure model-free account. Greater values indicate that the model-based account captures participants’ behaviour better than the model-free account.

## Results

### Sample and task behaviour

**Fig. 1:**
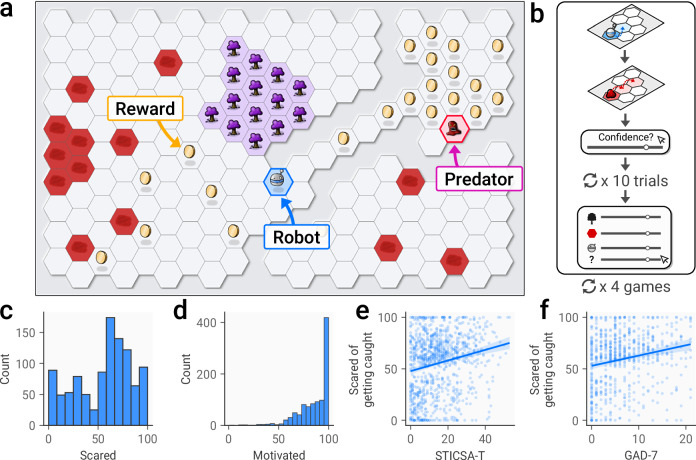
Task design and subjective ratings. **a** An overview of the task setup, where participants controlled a character (the robot) seeking rewards (the gold coins) while avoiding getting caught by a predator (a blob monster). The environment included two additional features: trees, shown in purple, and patches of red ground. Some areas of the environment were not accessible (areas with no pale hexagonal cells), providing barriers between different sections. **b** On each trial, subjects first moved the robot character. They were then asked to predict where they expected the predator to move, and were subsequently asked to report their confidence in this prediction. This repeated 10 times, before they were finally asked to report estimates of the predator’s preferences, along with their confidence in these estimates. After this the game ended, with subjects playing 4 games in total. **c** Responses to the question: “How scared were you of getting caught by the blob monster?”. **d** Responses to the question: “How motivated were you to avoid getting caught by the blob monster?”. **e** Correlation between trait anxiety (STICSA-T) scores and fear ratings in the game. **f** Correlation between state anxiety (GAD-7) and fear ratings.

A total of 1279 subjects (mean (SD) age = 34.34 (12.27); 571 male, 694 female, 11 other, 3 not reporting gender) completed a battery of questionnaires measuring aspects of mental health problems and neurodevelopmental conditions, with a subset of 1025 (mean (SD) age = 36.23 (12.31); 509 male, 507 female, 7 other, 3 did not report gender) also completing the predator-prey task (figs. 1a and 1b). After removing 9 participants with incomplete data and 20 subjects who failed questionnaire-based attention checks (see Methods), the final sample for analyses consisted of 996 subjects (mean (SD) age = 36.26 (12.28); 495 male, 494 female, 4 other, 3 not reporting gender).

To confirm that participants were motivated to perform the task, we asked them to report how scared they were of getting caught by the predator and how motivated they were overall to perform well. Mean ratings for both questions were high (*scared* mean (SD) = 55.66 (28.38), fig. 1c; *motivated* mean (SD) = 86.33 (15.73), fig. 1d; 0–100 scales), indicating that the task induced the expected affective state and that subjects were highly motivated to perform well. Notably, as found in prior work [37], ratings of task-induced anxiety were positively correlated with both trait (β = 0.38 [0.22, 0.54], *p* < 0.001, fig. 1e) and state (β = 0.86 [0.25, 1.48], *p* = 0.006, fig. 1f) anxiety. Since the task was designed to be straightforward (assuming participants correctly understood the predator’s goal), only 3.63% of participants were ever caught by the predator.

### Mental health and neurodevelopmental measures are structured hierarchically

We used exploratory factor analysis (EFA) to reveal the hierarchical structure of a range of measures used to assess features of mental health problems and neurodevelopmental conditions, chosen purposefully to cover a variety of dimensions proposed in treatments of hierarchical structure within psychopathology [22]. The hierarchical EFA procedure involved repeating the EFA procedure with increasing numbers of factors, up to a point determined by visual inspection of the scree plot and interpretability of resulting factors.

**Fig. 2:**
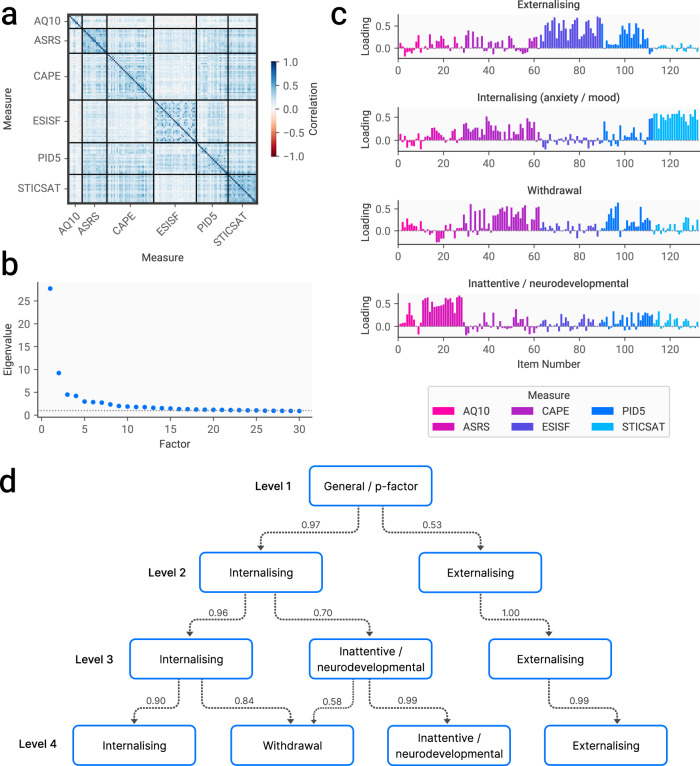
Hierarchical structure of mental health and neurodevelopmental dimensions. **a** Correlation matrix showing Pearson correlations between items in each measure. **b** Scree plot from the exploratory factor analysis. **c** Item loadings for the four factor solution. **d** Hierarchical structure of symptom dimensions, with values representing Pearson correlations between scores on one level and those on the level below. Correlations below 0.5 are not shown. AQ10 Autism Spectrum Quotient; ASRS Adult ADHD Self-Report Scale; CAPE Community Assessment of Psychic Experiences; ESISF Externalising Symptom Inventory Short Form; PID-5 Personality Inventory for DSM-5; STICSAT State Trait Inventory of Cognitive and Somatic Anxiety Trait scale.

There were strong correlations both within and between measures (fig. 2a). The EFA procedure resulted in up to four factors being retained (fig. 2b & c), which were related to one another hierarchically as determined according cross-level correlations (fig. 2d). At the highest level was a general psychopathology factor (akin to the p-factor), which split into internalising and externalising dimensions at the second level. At the third level, the internalising dimension split into an internalising dimension and an inattentive/neurodevelopmental dimension. Finally, at the fourth level, the internalising dimension split into a narrower internalising dimension focused on mood and anxiety and a withdrawal dimension. Levels beyond this resulted in solutions with uninterpretable factors. Loadings for each of these factors were clustered on relevant measures to an extent (for example, trait anxiety for the internalising dimension), but included strong loadings across a range of measures (fig. 2c).

### Externalising and inattentive/neurodevelopmental dimensions are differentially linked to inferences about the predator

**Fig. 3:**
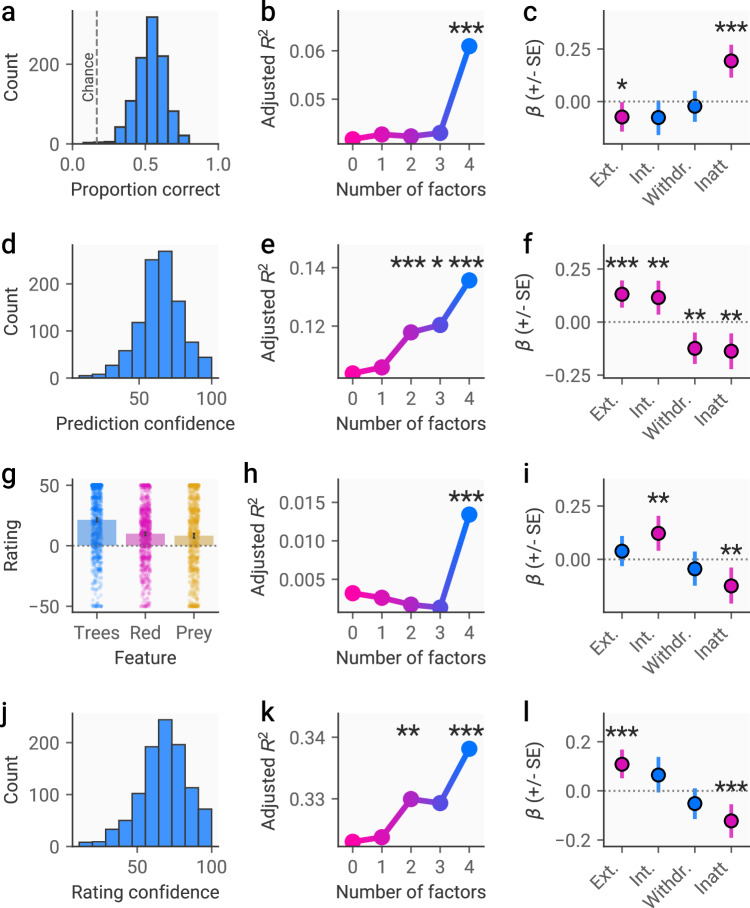
Associations between behaviour and symptom dimensions. **a** Histogram showing accuracy in predicting the predator’s movements across subjects. The chance level is 1/6. **b** results of sequential regression procedure showing adjusted *R*^2^ for models including symptom dimensions of increasing granularity, where the dependent variable is the proportion of correct responses. The X-axis represents the number of factors included in the model (i.e., the level of the hierarchy), where 0 represents covariates only. Significance is determined based on the change in model fit when moving a step down the hierarchy. **c** Regression coefficients from the model with the highest adjusted *R*^2^ when predicting the proportion of correct predictions. **d** Histogram showing confidence in movement prediction across subjects. **e** Results of sequential regression analyses for confidence in predictions. **f** Regression coefficients for the winning model predicting confidence in predictions. **g** Subjects’ ratings of the predator’s preference at the end of the task (the true preference was for the trees). **h** Results of sequential regression analyses predicting preference rating accuracy. **i** Regression coefficients for the winning model predicting preference rating accuracy. **j** Distribution of confidence in preference ratings across subjects. **k** Results of sequential regression analyses predicting confidence in preference ratings. **l** Regression coefficients for winning model predicting confidence in preference ratings. * = *p* < 0.05; ** = *p* < 0.01; *** = *p* < 0.001.

Results showed that participants were accurate in predicting the predator’s movements and performed significantly better than would be expected by chance (mean (SD) correct = 54% (10%), *t*(998) = 113.99, *p* < 0.001, *d* = 7.21, fig. 3a). Regression models including only lower levels of the hierarchy did not explain variance in prediction accuracy significantly better than a base model including only covariates. However, including the fourth level of the hierarchy (internalising, externalising, withdrawal, inattentive/neurodevelopmental) resulted in a significant increase in variance explained (adjusted *R*^2^ = 0.061 vs 0.043, *F*(1, 975) = 19.64, *p* < 0.001, fig. 3b). Examining the coefficients of this model revealed that externalising was linked to less accurate predictions (β = −0.07 [−0.14, −0.004], *p* = 0.04, fig. 3c), while the inattentive/neurodevelopmental dimension was linked to more accurate predictions (β = 0.19 [0.12, 0.28], *p* < 0.001, fig. 3c). Cross-validated permutation testing indicates that the model was able to explain variance in unseen data successfully (*R*^2^ = 0.04, *p* < 0.001). There was also no significant correlation between accuracy and incorrect responses to infrequency items when including all participants (*r* = −0.04, *p* = 0.21).

We followed this by examining how these dimensions related to reported confidence in predictions about the predator’s behaviour (fig. 3d). We also included accuracy of predictions in the model, to ensure that effects on confidence were not confounded by performance. This again revealed that variation in behaviour was best explained by level 4 of the hierarchy, with this model explaining significantly more variance than the level 3 model (adjusted *R*^2^ = 0.061 vs 0.043, *F*(1, 974) = 15.75, *p* < 0.001, fig. 3e), although simpler models also explained more variance than covariates alone (Table S3). This model was also significant when evaluated using cross-validated permutation testing (*R*^2^ = 0.11, *p* < 0.001). Notably, the inattentive/neurodevelopmental dimension was associated with reduced confidence (β = −0.14 [−0.21, −0.06], *p* < 0.001, fig. 3f), demonstrating a divergence between objective and subjective performance. Withdrawal also showed a negative relationship with confidence (β = −0.12 [−0.20, −0.05], *p* = 0.001, fig. 3f), while there were positive associations with internalising (β = 0.13 [0.07, 0.20], *p* < 0.001, fig. 3f), and externalising (β = 0.12 [0.04, 0.20], *p* = 0.005, fig. 3f). Notably, we also found significant gender effects, where male participants made less accurate predictions and were less confident in their predictions (see Tables S7 and S8).

Participants were also asked to estimate the predator’s preference for each of the three features in the environment (trees, red ground, the robot). On average, participants were significantly more accurate in predicting the predator’s true preference for the trees than would be expected by chance (*t*(998) = 113.99, *p* < 0.001, *d* = 7.21, fig. 3g). Using the sequential modelling approach described above, level 4 of the hierarchy best explained the error of these reports (adjusted *R*^2^ = 0.01 vs 0.00, *F*(1, 975) = 12.95, *p* < 0.001, fig. 3h & i) and confidence in their accuracy (adjusted *R*^2^ = 0.34 vs 0.33, *F*(1, 975) = 13.99, *p* < 0.001, fig. 3j & k). Both models were significant in cross-validation (error: *R*^2^ = −0.027, *p* = 0.004; confidence: *R*^2^ = 0.32, *p* < 0.001). We again observed a divergence in objective and subjective performance linked to inattentive/neurodevelopmental symptoms, whereby higher scores on this dimension were linked to reduced error (β = −0.13 [−0.21, −0.04], *p* = 0.002, fig. 3i) but lower confidence (β = −0.12 [−0.19, −0.06], *p* < 0.001, fig. 3l). We also found that higher levels of internalising symptoms were linked to greater error (β = 0.12 [0.04, 0.21], *p* = 0.005, fig. 3i), while externalising symptoms were linked to greater confidence in these ratings (β = 0.11 [0.05, 0.17], *p* < 0.001, fig. 3l). We further investigated biases in these reports, focusing on ratings of the predator’s preferences for the prey, to explore whether certain dimensions may be associated with a tendency to overestimate the predator’s preference for the participant’s character specifically. However, this was not significantly linked to any level of the hierarchy. We found no effects of gender on rating accuracy, although male participants reported lower confidence (Tables S9 and S10).

### Greater accuracy associated with inattentive/neurodevelopmental characteristics is explained by preferential use of model-based inference

We next sought to uncover the mechanisms underpinning variation in task performance using a modelling approach developed in our prior work [2] that involved building computational models to explain participants’ predictions of the predator’s behaviour based on either model-free learning or model-based planning.

**Fig. 4:**
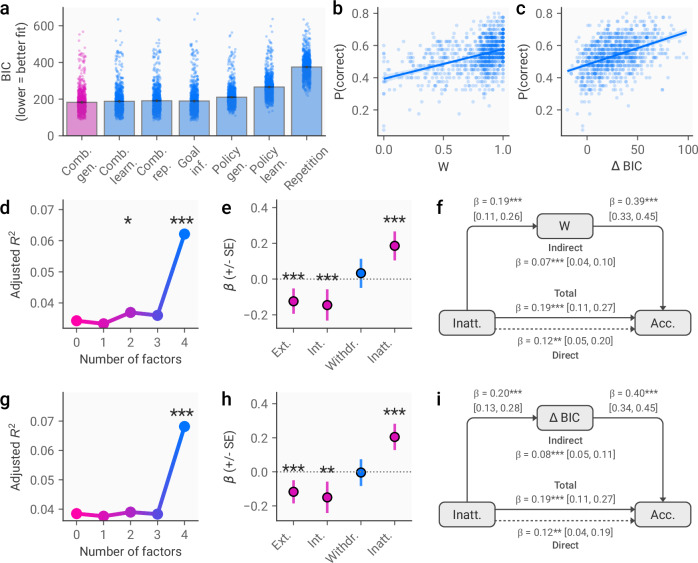
Computational modelling of movement prediction behaviour and links to transdiagnostic dimensions. **a** Model fit, indicating that a model that combines model-free and model-based strategies (the combined generalisation model, see Methods) provides the best fit to the data. **b** Correlation between model-based weighting parameter from the winning model (W) and accuracy in the task. Higher *W* values represent greater use of model-based strategies. **c** Association between relative fit of models using model-free and model-based strategies and accuracy in the task. Higher values of ΔBIC represent greater fit of the model-based implementation, and hence greater use of a model-based strategy. **d** Results of sequential regression procedure showing adjusted *R*^2^ for models including symptom dimensions of increasing granularity, where the dependent variable is the weighting parameter *W*. **e** Coefficients from winning regression model predicting weighting parameter values. **f** Result of mediation analysis examining mediation of the link between inattentive/neurodevelopmental dimension scores and accuracy in the task by weighting parameter values. Indirect path Inattentive scores > *W* > accuracy. Direct path - Inattentive/neurodevelopmental scores > accuracy, controlling for *W* scores. Total path = Inattentive/neurodevelopmental scores > accuracy, without controlling for *W* scores. **g** Results of sequential regression analyses where the difference in model fit between model-based and model-free implementations is the dependent variable. **h** Coefficients from the winning regression model predicting difference in model fit. **i** Results of mediation analysis, as described in F) but with the difference in model fit as the mediator. BIC Bayesian Information Criterion, * = *p* < 0.05; ** = *p* < 0.01; *** = *p* < 0.001.

Model comparison revealed that a combination of model-based planning and model-free learning provided the best fit to the data, accounting for model complexity using the Bayesian Information Criterion (BIC, fig. 4a). Parameter estimates from this model showed that the balance was skewed in favour of model-based planning, as indicated by high *W* values (mean (SD) = 0.78 (0.23), higher = more model-based). Higher accuracy in the task was associated with both better relative fit of the model-based strategy (ρ(982) = 0.40, *p* < 0.001, fig. 4b) and greater weight of the model-based component of the combined model (r(982) = 0.42, *p* < 0.001, fig. 4c). When examining reaction times, we found a suggestive pattern of faster responding in participants who used model-based strategies, although the task was not optimised for collection of reaction times (see Supplementary Material).

When examining the extent to which variation in model parameters was explained by hierarchical dimensions of mental health and neurodevelopmental characteristics, we found again that a model incorporating level 4 of the hierarchy best explained variation in the model-based weight parameter *W* (adjusted *R*^2^ = 0.07 vs 0.04, *F*(1, 975) = 32.27, *p* < 0.001, figs. 4d, e & f), alongside the difference in model fit between model-based and model-free implementations (adjusted *R*^2^ = 0.06 vs 0.04, *F*(1, 975) = 28.22, *p* < 0.001, fig. 4g). Cross-validated permutation testing confirmed that both of these models predicted a significant amount of variance in unseen data (*W* parameter: *R*^2^ = 0.05, *p* < 0.001; model fit difference: *R*^2^ = 0.05, *p* < 0.001). Model coefficients indicated that this was a result of a divergence between different dimensions; greater use of a model-based strategy was associated with the inattentive/neurodevelopmental dimension (*W*: β = 0.19 [0.11, 0.26], *p* < 0.001, fig. 4e; model fit: β = 0.20 [0.13, 0.28], *p* < 0.001, fig. 4h), whereas reduced use of the model-based strategy was linked to both internalising (*W*: β = −0.15 [−0.23, −0.06], *p* = 0.001, fig. 4e; model fit: β = −0.15 [−0.23, −0.07], *p* < 0.001, fig. 4h) and externalising (*W*: β = −0.12 [−0.19, −0.06], *p* < 0.001, fig. 4e; model fit: β = −0.12 [−0.19, −0.05], *p* = 0.001, fig. 4h) dimensions. We also found a significant gender effect, where male participants were less model-based according to both measures (Tables S14 and S15).

This suggested that the observed association between the inattentive/neurodevelopmental dimension and improved accuracy in the task may have been mediated by greater use of a model-based strategy. To test this formally, we conducted mediation analyses with both indices of model-based decision-making (the *W* parameter value and the difference in model fit between model-free and model-based implementations). This demonstrated that in both cases, both the indirect and direct paths were significant, suggesting that the link between inattentiveness and accuracy was partially mediated by using model-based strategies in the task (fig. 4f &i, Tables S16 & S17). We also observed a similar pattern when performing mediation analysis with the externalising dimension, although here the relationship between externalising and poorer predictions was fully mediated by the use of model-based strategies (Tables S18 & S19).

## Discussion

Flexible decision-making is key to our ability to navigate complex, interactive environments in the real world, and represents a promising candidate for understanding how these processes are affected by mental health and neurodevelopmental conditions. We sought to determine its specific role using a validated predator-prey task combined with computational modelling of the mechanisms underlying behaviour, taking an exploratory approach to identify promising avenues for future research. Broadly, our results are in line with suggestions that inattentive symptoms may reflect selective attention based on internal motives [38].

One of the most consistent findings to emerge was that people higher in inattentive/neurodevelopmental symptoms performed better in predicting the predator’s behaviour and inferring its goals, despite showing reduced confidence in their ability to do so. Computational modelling revealed that this was explained by the greater use of more demanding model-based strategies to predict the predator’s next moves, suggesting that people scoring higher on this dimension are either more able to use these strategies or simply do so more readily. Importantly, while it is necessary to be cautious in interpreting any result that could be influenced simply by an inability to attend to the task as required, the fact that greater inattentive/neurodevelopmental traits were linked to *higher* accuracy indicates that this is not an artifact of inattention within the task. Our use of hierarchical transdiagnostic dimensions also enabled us to show that this is not an effect of general psychopathology, or a higher order internalising/externalising dimension, but is instead specific to lower-level dimensions.

While perhaps counterintuitive on the surface, this finding may reflect neglected characteristics of individuals who may typically be described as “inattentive”. Some evidence suggests that inattentive symptoms, as commonly seen within attention deficit hyperactivity disorder (ADHD), may reflect a more selective deployment of demanding cognitive resources towards motivationally salient tasks rather than a general deficit in attention [38]. Our results are in line with this proposal, suggesting that people higher in these traits may be more likely to use computationally complex, model-based strategies to inform decision-making in complex environments. While links between model-based planning and inattentive traits are not a well-reported phenomenon, these neurodevelopmental traits are often not assessed in transdiagnostic symptom batteries [20, 39], meaning that this may simply be due to their omission from prior studies. More generally, aspects of decision-making behaviour have been found to be impaired in ADHD, where inattentive/neurodevelopmental characteristics are most prominent [40–42]. However, these processes are typically assessed using highly artificial tasks (e.g., go/no-go tasks) [43], and there is evidence that goal-directed behaviour may be enhanced in more real-world situations [44]. Future efforts in this area may include debriefings that specifically enquire about the extent to which participants report their attention in the task being dependent on motivation.

We also found that externalising symptoms were consistently associated with an opposite pattern of behaviour, with those scoring higher on this dimension making more errors when predicting behaviour. Contrasting with the inattentive/neurodevelopmental dimension, this was explained by a reduced tendency to use model-based strategies and a reliance on simpler but less accurate error-driven learning. This may suggest that individuals higher in this dimension are more likely to draw false inferences about others’ behaviour, but be overly confident in these incorrect inferences. This is reminiscent of findings demonstrating impairments in social inference skills, such as facial emotion recognition [45] and a general reduction in attention to socially-relevant cues [46], in people with externalising disorders, but extends these findings to more complex interactive decision problems. Intriguingly, we also found a similar pattern of results with regard to internalising symptoms in predicting the predator’s preferences, where people scoring higher on this dimension made more errors but were more confident about their ratings. This may suggest that people higher in this dimension are more likely to draw false, but certain, inferences about others’ intentions, and is in line with prior observations of impaired theory of mind in depression [47] and social anxiety [48]. We also observed that male participants were less likely to rely on model-based strategies, as we have seen in prior work [49]

The factors we derive from our questionnaire battery are comparable to an extent with those identified in prior studies. For example, the dimensions we identify mostly align with those found in our prior work. While comparison with other studies assessing different symptom domains is challenging, we do find that a general factor at the highest level can be divided into externalising and internalising dimensions, a common finding in similar studies [33]. Our results begin to diverge from the majority of studies as we move down the hierarchy, primarily due to the emergence of an inattentive/neurodevelopmental factor, which is not often seen [50]; we believe this is simply because these traits are often not measured in batteries of mental health symptoms [50], despite the fact that characteristics of neurodevelopmental conditions are transdiagnostic and associated with symptoms of mental health problems [23]. Indeed, our findings are consistent with a growing number of studies that did include neurodevelopmental symptoms alongside other symptoms [23]. In general, it is challenging to compare the more specific factors identified here to previous studies that use quite different assessment batteries, since the questionnaires included will determine the factors identified. Nevertheless, our findings are broadly consistent with studies testing a similar set of symptoms, indicating that these results are robust to differences in assessment.

One question raised by these findings is why the use of model-based strategies varies according to these transdiagnostic dimensions. It is possible that this may reflect variability in the neural systems underpinning these processes given that regions known to be involved in prospective planning, such as the hippocampus [51–53], are implicated in various mental health and neurodevelopmental problems [54, 55]. However, a tendency to adopt a model-based approach may also be influenced by perceptions of the benefit of doing so [14], or from other ongoing states related to mental health such as stress [56] or cognitive impairment [57]. It is also possible that such individual differences emerge due to experiences, whether challenging or positive, during developmental periods where model-based decision processes are coming online [58, 59]. A further consideration is that, while our findings are consistent with the use of model-based strategies, participants may also be using simpler approximations such as the successor representation [60].

We also note that while we address a common question in the field of computational psychiatry, namely how the preferential use of model-based over model-free strategies is associated with mental health problems, we do so in a setting that differs substantially from prior work. Rather than focusing on the individual’s own reward or threat-guided decision-making directly, as is commonly assessed using tasks such as the “two-step” task [12, 20, 49], we examine how individuals use these strategies to infer another agent’s likely behaviour. While we are hesitant to call this a “social” inference task given that participants do not believe they are playing against another human, this is more aligned with studies of belief inference in social settings [3, 61].

While our task has advantages in that it is more naturalistic and promotes the use of more complex decision-making strategies than traditional tasks [12, 62], there are also limitations. The task was highly structured, following a turn-based format, which is less reflective of naturalistic decision problems. Furthermore, the predator had a simple goal and there was relatively little unpredictability in its behaviour assuming this goal was known; this is important given the proposed role of uncertainty-related computations in conditions such as anxiety [37, 63, 64]. Its goal also did not change over time, and it did not perform any higher-order planning operations accounting for its own beliefs about the subjects’ beliefs. Nonetheless, we believe that the task is capable of evoking decision processes that better approximate those deployed in the real world than many simpler tasks [65]. We also cannot be certain of whether our findings are specific to threat-related inference, since our task did not include a non-threatening condition. Future work could examine the extent to which these processes are associated with symptom dimensions in situations that do not involve threat. Finally, our task was not designed to capture accurate reaction times, which could have provided an additional window into the strategies used by participants.

Likewise, our assessment of mental health symptoms and neurodevelopmental characteristics has limitations, despite our efforts to provide a broad characterisation of transdiagnostic dimensions and their hierarchical structure. Most notably, our sample was recruited online from the general population. While some of these participants would likely have clinically significant mental health problems or neurodevelopmental conditions, this was not an explicit inclusion criterion and the majority of the sample will sit within the subclinical or typical range.

Furthermore, due to the paucity of research in this area, we elected to take a purely exploratory and hypothesis generating approach here; as such, our results require replication; this was not undertaken due to practical and financial considerations, since the required sample size would be large. Nonetheless, our large sample, along with evidence from cross-validation that our results have the potential to generalise, provide some confidence in the relationships we identify. We note, however, that our cross-validation analyses are not a substitute for true replication, and are likely to overstate the true explanatory power of our models relative to what may be observed in a new sample. Finally, the *R*^2^ values within our cross-validation analyses are small, or even negative (potentially indicating predictions that are correlated with the true value but systematically biased), but this is not entirely surprising given the multifactorial nature of mental health, where any one variable is unlikely to explain a great amount of variance. These analyses demonstrate statistical significance, but not practical utility in terms of symptom prediction.

Our results highlight the value of considering decision-making beyond simple reward-guided choice tasks and, likely due to our focus on the processes supporting decision-making within a complex, interactive situation, do not necessarily mirror those from traditional decision-making tasks such as the two-step task [12, 16, 49]. This is an avenue that future work could explore in more depth, particularly in more explicitly social scenarios. Furthermore, while our results do not have immediate clinical implications, they do indicate that it is important to consider symptoms in context. We show that individuals who report difficulties with attention in fact perform better within this task. This suggests that certain symptoms may be highly context-dependent, and is in line with prior work demonstrating that individuals with ADHD primarily demonstrate performance deficits in more tedious and unengaging tasks [66–68].

## Supplementary information

Supplemental material

## Data Availability

Code is available on GitHub at https://github.com/tobywise/interactive-avoidance-mental-health_public and all data is available through the Open Science Framework at https://osf.io/a7e3k/.

## Code availability

Code is available on GitHub at https://github.com/tobywise/interactive-avoidance-mental-health_public and all data is available through the Open Science Framework at https://osf.io/a7e3k/.
