# Supplementary material for: Transdiagnostic mental health symptom dimensions predict use of flexible model-based inference in complex environments
Source: Transl Psychiatry. 2026 Mar 7;16:141. doi: 10.1038/s41398-026-03922-w (PMC12982692; doi:10.1038/s41398-026-03922-w)
Supplement: Supplementary file 1 — Supplemental material [file 41398_2026_3922_MOESM1_ESM.pdf]

Supplementary Results

Analysis of reaction times

While our task was not well designed for assessing reaction times, we conducted some preliminary analyses investigating whether reaction times when predicting the predator's movement were associated with the use of model-based strategies across participants, assuming that model-based computations may require additional time relative to model-free learning. Reaction times were cleaned (removing responses <300ms and >10s, as well as those +/- 3SD from the mean for each participant) and then log transformed. Unexpectedly, we found no significant correlations between reaction times when predicting the predator's initial move and the weighting parameter from our model (r = -0.05, p = 0.10). The same pattern emerged when using the relative model fit between pure model-based and model-free models (r = -0.06, p = 0.08).

We did, however, find a significant negative correlation when examining predictions of the predator's second move (weighting parameter: r = -0.11, p < .001; model fit difference: r = - 0.14, p < .001), although these effects were weak. Since such effects could be explained by inattention, whereby inattentive participants both take longer to respond and appear less model-based, we performed the same analyses using reaction times for the participant's own movements. Any effect driven purely by inattention should also emerge here, despite the fact that these decisions do not rely on the same model-based computations. Here, we found no significant correlations (weighting parameter: r = 0.01, p = 0.69; model fit difference: r = 0.01, p = 0.72), suggesting that these results were not driven by inattention.

Sensitivity analyses

In our primary analyses, we included participants who failed a single infrequency item. To provide additional evidence that our results were not driven by inattention, we repeated our analyses including only participants who passed every attention check and infrequency item (N=913). The results were unchanged, with the exception of an effect of externalising symptoms on prediction accuracy, showing a small effect in our main analyses, which was no longer significant (Figure S2 and S3).

Supplementary Figures

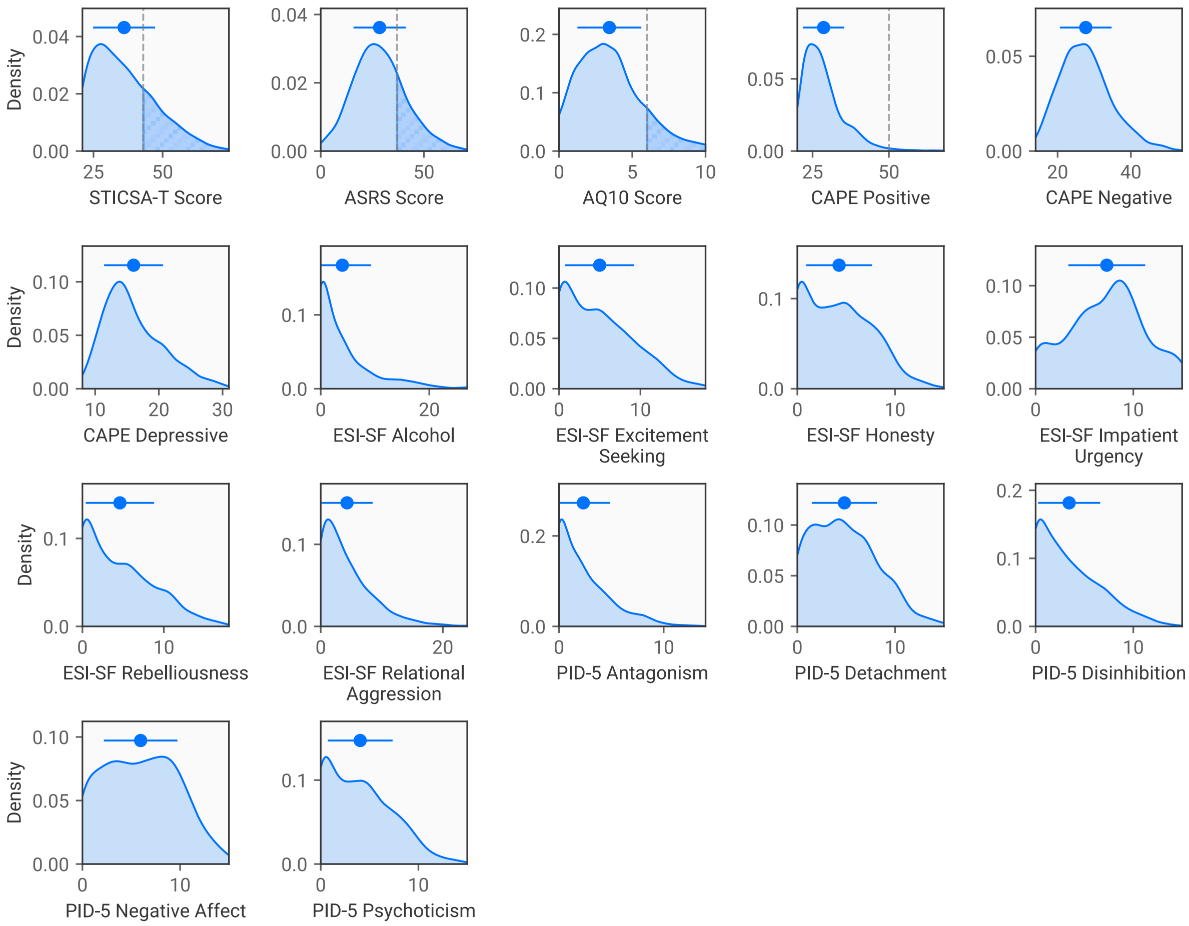

Figure S1. Distributions of responses to questionnaires within the subsample of participants who completed the behavioural task, excluding participants who did not meet our criteria for inclusion based on attention checks. For measures where cutoffs for clinically significant symptoms have been established (STICSA-T[1], ASRS[2], AQ-10[3], CAPE positive subscale[4]), the shaded area represents participants scoring above this cutoff. AQ10 = Autism Spectrum Quotient; ASRS = Adult ADHD Self-Report Scale; CAPE = Community Assessment of Psychic Experiences; ESISF = Externalising Symptom Inventory Short Form; PID-5 = Personality Inventory for DSM-5; STICSAT = State Trait Inventory of Cognitive and Somatic Anxiety Trait scale.

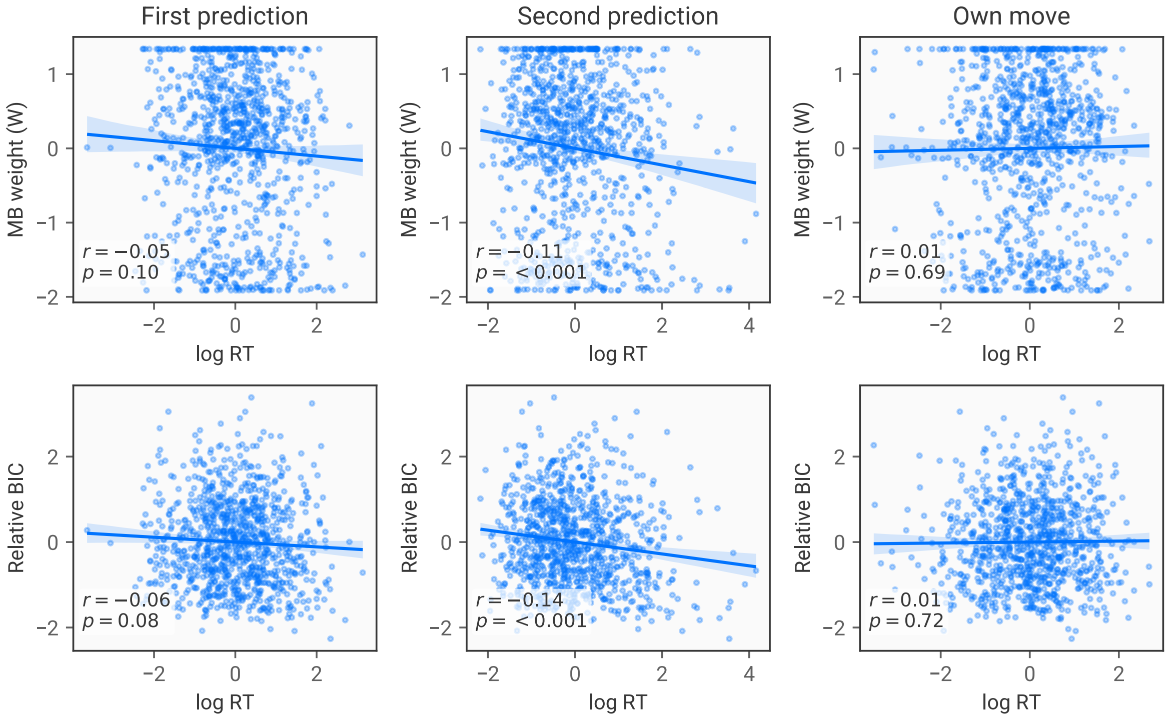

Figure S2. Correlations between use of model-based strategies for predicting the predator's moves and reaction times when making predictions. The upper row represents correlations using the weighting parameter from a combined model-based and model-free model as an indexed of model-based predictions, while the bottom row uses the difference in model fit between pure model-based and model-free fits. The left column represents the first prediction made by the participant, the second column represents the second prediction, and the third column represents the participant's own move.

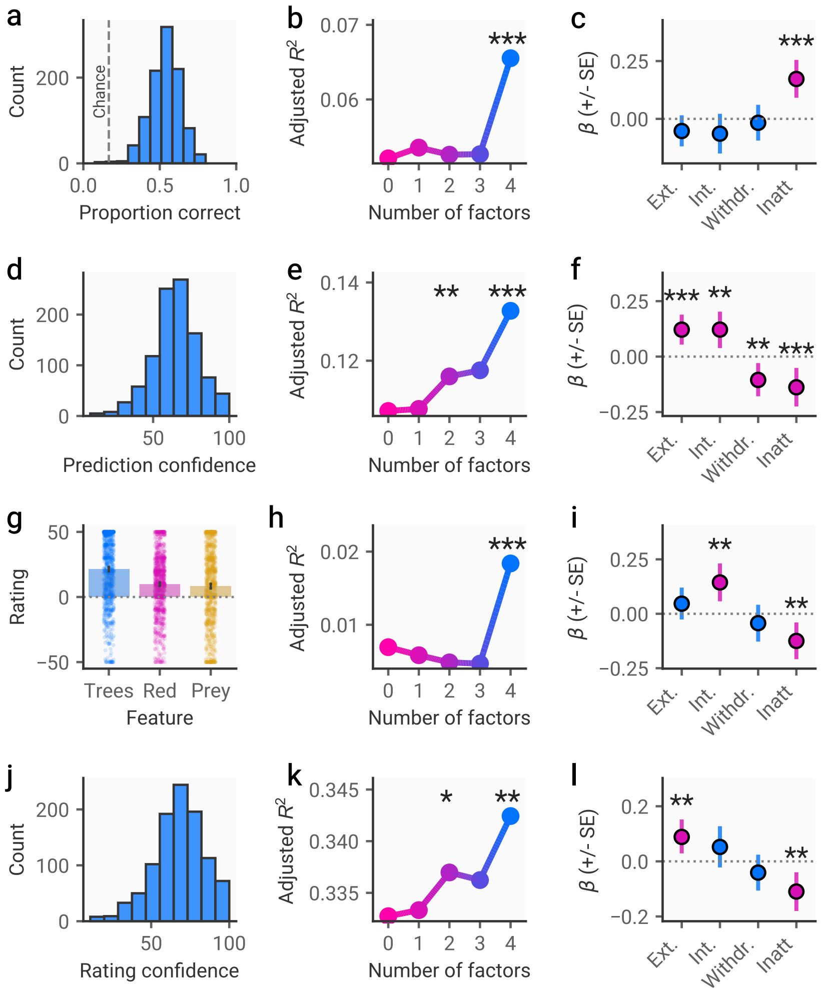

Figure S3. Associations between behaviour and symptom dimensions when including only participants who pass all attention checks and infrequency items. A) Histogram showing accuracy in predicting the predator's movements across subjects. The chance level is 1/6. B) results of sequential regression procedure showing adjusted R^2 for models including symptom dimensions of increasing granularity, where the dependent variable is the proportion of correct responses. The X-axis represents the number of factors included in the model (i.e., the level of the hierarchy), where 0 represents covariates only. Significance is determined based on the change in model fit when moving a step down the hierarchy. C) Regression coefficients from the model with the highest adjusted R^2 when predicting the proportion of correct predictions. D) Histogram showing confidence in movement prediction across subjects. E) Results of sequential regression analyses for confidence in predictions. F) Regression coefficients for the winning model predicting confidence in predictions. G) Subjects' ratings of the predator's preference at the end of the task (the true preference was for the trees). H) Results of sequential regression analyses predicting preference rating accuracy. I) Regression coefficients for the winning model predicting preference rating accuracy. J) Distribution of confidence in preference ratings across subjects. K) Results of sequential regression analyses predicting confidence in preference ratings. L) Regression coefficients for winning model predicting confidence in preference ratings. * = $p < .05$; ** = $p < .01$; *** = $p < .001$.

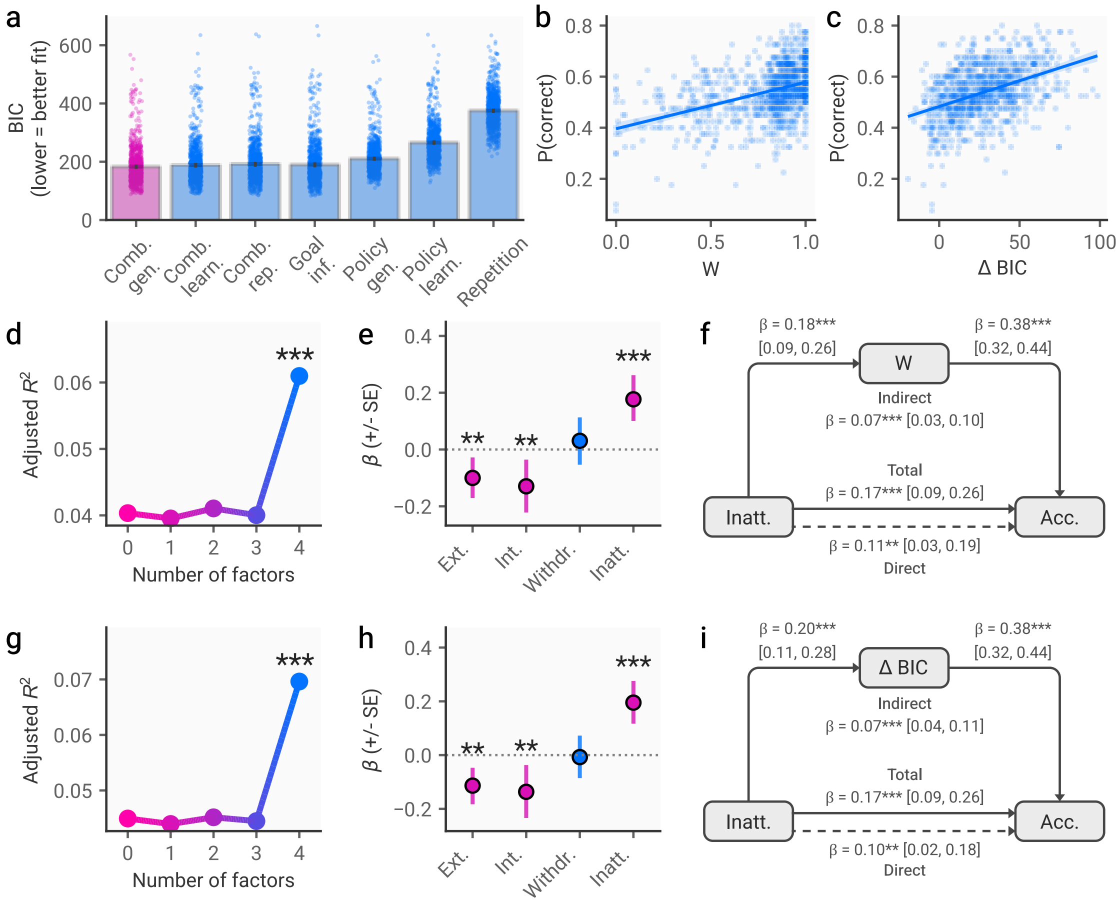

Figure S4. Computational modelling of movement prediction behaviour and links to transdiagnostic dimensions when including only participants who pass all attention checks and infrequency items. A) Model fit, indicating that a model that combines model-free and model-based strategies (the combined generalisation model, see Methods) provides the best fit to the data. B) Correlation between model-based weighting parameter from the winning model (W) and accuracy in the task. Higher *W* values represent greater use of model-based strategies. C) Association between relative fit of models using model-free and model-based strategies and accuracy in the task. Higher values of ΔBIC represent greater fit of the model-based implementation, and hence greater use of a model-based strategy. D) Results of sequential regression procedure showing adjusted R^2 for models including symptom dimensions of increasing granularity, where the dependent variable is the weighting parameter *W*. E) Coefficients from winning regression model predicting weighting parameter values. F) Result of mediation analysis examining mediation of the link between inattentive/neurodevelopmental dimension scores and accuracy in the task by weighting parameter values. Indirect path = Inattentive scores > *W* > accuracy. Direct path - Inattentive/neurodevelopmental scores > accuracy, controlling for *W* scores. Total path = Inattentive/neurodevelopmental scores > accuracy, without controlling for *W* scores. G) Results of sequential regression analyses where the difference in model fit between model-based and model-free implementations is the dependent variable. H) Coefficients from the winning regression model predicting difference in model fit. I) Results of mediation analysis, as described in F) but with the difference in model fit as the mediator. BIC = Bayesian Information Criterion, * = p < .05; ** = p < .01; *** = p < .001.

Supplementary Tables

df_R	SS_R	SS_{diff}	F	p
979	938.15	-	-	-
978	936.24	1.92	2.04	0.153
977	935.67	0.57	0.61	0.435
976	934.01	1.65	1.76	0.185
975	915.57	18.44	19.64	**<.001**

Table S1. Model comparison results for models of subjects' predictions of the predator's movements. df_R = residual degrees of freedom, SS_R = residual sum of squares, SS_{diff} = difference in residual sum of squares between models, F = F-statistic, p = p-value.

df_R	SS_R	SS_{diff}	F	p
979	964.65	-	-	-
978	964.23	0.42	0.43	0.513
977	964.17	0.07	0.07	0.796
976	964.16	0.00	0.00	0.967
975	962.72	1.44	1.46	0.227

Table S2. Model comparison results for models of subjects' reaction times when predicting the predator's movements df_R = residual degrees of freedom, SS_R = residual sum of squares, SS_{diff} = difference in residual sum of squares between models, F = F-statistic, p = p-value.

df_R	SS_R	SS_{diff}	F	p
978	876.51	-	-	-
977	873.60	2.91	3.36	0.067
976	861.00	12.60	14.58	**<.001**
975	857.66	3.34	3.86	**0.050**
974	841.91	15.75	18.22	**<.001**

Table S3. Model comparison results for models of subjects' confidence in predictions of the predator's movements. df_R = residual degrees of freedom, SS_R = residual sum of squares, SS_{diff} = difference in residual sum of squares between models, F = F-statistic, p = p-value.

df_R	SS_R	SS_{diff}	F	p
979	975.87	-	-	-
978	975.45	0.41	0.42	0.517
977	975.32	0.13	0.13	0.717
976	974.70	0.62	0.63	0.428
975	961.92	12.78	12.95	**<.001**

Table S4. Model comparison results for models of subjects' ratings of the predator's preferences. df_R = residual degrees of freedom, SS_R = residual sum of squares, SS_{diff} = difference in residual sum of squares between models, F = F-statistic, p = p-value.

df_R	SS_R	SS_{diff}	F	p
976	667.85	-	-	-
975	666.43	1.42	2.12	0.146

df_R	SS_R	SS_{diff}	F	p
974	659.64	6.79	10.15	**0.001**
973	659.61	0.03	0.04	0.833
972	650.25	9.36	13.99	**<.001**

Table S5. Model comparison results for models of subjects' confidence in ratings of the predator's preferences. df_R = residual degrees of freedom, SS_R = residual sum of squares, SS_{diff} = difference in residual sum of squares between models, F = F-statistic, p = p-value.

df_R	SS_R	SS_{diff}	F	p
979	981.33	-	-	-
978	980.99	0.35	0.35	0.556
977	981.00	-0.02	-0.02	1.000
976	980.98	0.03	0.03	0.873
975	978.43	2.55	2.54	0.111

Table S6. Model comparison results for models of subjects' ratings of the predator's preference for the robot character. df_R = residual degrees of freedom, SS_R = residual sum of squares, SS_{diff} = difference in residual sum of squares between models, F = F-statistic, p = p-value.

	β	β_{SE}	t	p	$CI_{2.5}$	$CI_{97.5}$
Intercept	0.12	0.05	2.59	**0.010**	0.03	0.21
Age	-0.16	0.03	-4.81	**<.001**	-0.22	-0.09
Gender	-0.24	0.07	-3.45	**0.001**	-0.38	-0.10
Motivation	0.08	0.03	2.60	**0.010**	0.02	0.14
Externalising	-0.07	0.04	-2.08	**0.038**	-0.14	-0.00
Internalising	-0.08	0.04	-1.80	0.071	-0.16	0.01
Withdrawal	-0.02	0.04	-0.58	0.560	-0.10	0.05
Inattention	0.19	0.04	4.82	**<.001**	0.12	0.27

Table S7. Coefficients for winning model of subjects' predictions of the predator's movements. β = regression coefficient, β_{SE} = standard error of regression coefficient, t = t-statistic, p = p-value, $CI_{2.5}$ = 95% confidence interval lower bound, $CI_{97.5}$ = 95% confidence interval upper bound.

	β	β_{SE}	t	p	$CI_{2.5}$	$CI_{97.5}$
Intercept	0.07	0.04	1.61	0.108	-0.02	0.16
Age	-0.00	0.03	-0.02	0.986	-0.06	0.06
Gender	-0.14	0.07	-2.14	**0.033**	-0.28	-0.01
Prop. correct	0.17	0.03	5.56	**<.001**	0.11	0.23
Motivation	0.24	0.03	7.89	**<.001**	0.18	0.29
Externalising	0.13	0.03	3.86	**<.001**	0.07	0.20
Internalising	0.12	0.04	2.84	**0.005**	0.04	0.20
Withdrawal	-0.12	0.04	-3.36	**0.001**	-0.20	-0.05
Inattention	-0.14	0.04	-3.53	**<.001**	-0.21	-0.06

Table S8. Coefficients for winning model of subjects' confidence in predictions of the predator's movements. β = regression coefficient, β_{SE} = standard error of regression coefficient, t = t-statistic, p = p-value, $CI_{2.5}$ = 95% confidence interval lower bound, $CI_{97.5}$ = 95% confidence interval upper bound.

	β	β_{SE}	t	p	$CI_{2.5}$	$CI_{97.5}$
Intercept	-0.04	0.05	-0.74	0.458	-0.13	0.06
Age	0.07	0.03	1.99	**0.047**	0.00	0.13
Gender	0.07	0.07	0.99	0.322	-0.07	0.21
Motivation	0.02	0.03	0.68	0.496	-0.04	0.08
Externalising	0.04	0.04	1.06	0.290	-0.03	0.11
Internalising	0.12	0.04	2.82	**0.005**	0.04	0.21
Withdrawal	-0.04	0.04	-1.13	0.259	-0.12	0.03
Inattention	-0.12	0.04	-3.03	**0.002**	-0.20	-0.04

Table S9. Coefficients for winning model of subjects' ratings of the predator's preferences. β = regression coefficient, β_{SE} = standard error of regression coefficient, t = t-statistic, p = p-value, $CI_{2.5}$ = 95% confidence interval lower bound, $CI_{97.5}$ = 95% confidence interval upper bound.

	β	β_{SE}	t	p	$CI_{2.5}$	$CI_{97.5}$
Intercept	0.10	0.04	2.43	**0.015**	0.02	0.17
Age	-0.02	0.03	-0.76	0.450	-0.08	0.03
Gender	-0.18	0.06	-3.08	**0.002**	-0.30	-0.07
|Error|	0.16	0.03	6.00	**<.001**	0.11	0.22
Rating	0.30	0.10	2.99	**0.003**	0.10	0.51
Rating2	-0.14	0.10	-1.35	0.177	-0.34	0.06
Motivation	0.22	0.03	8.20	**<.001**	0.17	0.27
Externalising	0.11	0.03	3.61	**<.001**	0.05	0.17
Internalising	0.06	0.04	1.79	0.073	-0.01	0.14
Withdrawal	-0.05	0.03	-1.59	0.113	-0.12	0.01
Inattention	-0.12	0.03	-3.60	**<.001**	-0.19	-0.06

Table S10. Coefficients for winning model of subjects' confidence in ratings of the predator's preferences. β = regression coefficient, β_{SE} = standard error of regression coefficient, t = t-statistic, p = p-value, $CI_{2.5}$ = 95% confidence interval lower bound, $CI_{97.5}$ = 95% confidence interval upper bound.

Parameter	Measure	β	β_{SE}	t	p	$CI_{2.5}$	$CI_{97.5}$	p_{FDR}
Prop. correct move predictions	AQ-10	0.01	0.03	0.41	0.674	-0.05	0.08	0.975
	STICSA-T	0.05	0.03	1.55	0.111	-0.01	0.11	0.555
	LSAS	0.00	0.03	0.03	0.975	-0.06	0.06	0.975
	PHQ-8	0.03	0.04	0.80	0.419	-0.05	0.11	0.975
	GAD-7	0.00	0.04	0.01	0.973	-0.08	0.08	0.975
Confidence in move predictions	AQ-10	0.10	0.03	3.36	<.001	0.04	0.17	**<.001**
	STICSA-T	-0.05	0.03	-1.65	0.095	-0.11	0.01	0.119
	LSAS	-0.11	0.03	-3.55	<.001	-0.17	-0.05	**<.001**
	PHQ-8	-0.10	0.04	-2.45	0.020	-0.18	-0.01	**0.033**
	GAD-7	-0.06	0.04	-1.46	0.152	-0.14	0.02	0.152
|Preference rating error|	AQ-10	0.02	0.03	0.72	0.513	-0.04	0.09	0.794
	STICSA-T	-0.01	0.03	-0.19	0.879	-0.07	0.06	0.879
	LSAS	-0.02	0.03	-0.51	0.635	-0.09	0.05	0.794
	PHQ-8	0.04	0.04	0.84	0.428	-0.06	0.13	0.794
	GAD-7	0.06	0.04	1.28	0.238	-0.03	0.14	0.794
Confidence in preference ratings	AQ-10	0.10	0.03	3.32	0.004	0.03	0.17	**0.020**
	STICSA-T	-0.05	0.03	-1.51	0.161	-0.11	0.02	0.161
	LSAS	-0.09	0.03	-2.79	0.012	-0.15	-0.02	**0.030**
	PHQ-8	-0.07	0.04	-1.87	0.089	-0.16	0.01	0.148
	GAD-7	-0.05	0.04	-1.41	0.131	-0.13	0.02	0.161
Prey rating	AQ-10	-0.01	0.03	-0.24	0.775	-0.07	0.06	0.985
	STICSA-T	0.01	0.03	0.22	0.843	-0.06	0.08	0.985
	LSAS	-0.00	0.03	-0.02	0.985	-0.07	0.06	0.985
	PHQ-8	0.06	0.04	1.50	0.139	-0.02	0.15	0.695
	GAD-7	0.04	0.04	1.03	0.317	-0.04	0.13	0.792

Table S11. Coefficients for additional measures predicting different aspects of behaviour, taken from regression models including the same covariates as the primary models. β = regression coefficient, β_{SE} = standard error of regression coefficient, t = t-statistic, p = p-value, $CI_{2.5}$ = 95% confidence interval lower bound, $CI_{97.5}$ = 95% confidence interval upper bound., AQ-10 = Autism Spectrum Quotient, STICSA-T = State Trait Inventory of Cognitive and Somatic Anxiety, LSAS = Liebowitz Social Anxiety Scale, PHQ-8 = Patient Health Questionnaire, GAD-7 = Generalized Anxiety Disorder Assessment

df_R	SS_R	SS_{diff}	F	p
979	941.34	-	-	-
978	941.25	0.09	0.10	0.752
977	938.92	2.33	2.50	0.114
976	938.62	0.30	0.32	0.573
975	908.55	30.07	32.27	**<.001**

Table S12. Model comparison results for regression models predicting relative fit of model-based and model-free models of movement prediction. df_R *= residual degrees of freedom,* SS_R *= residual sum of squares,* SS_{diff} *= difference in residual sum of squares between models,* F *= F-statistic,* p *= p-value.*

df_R	SS_R	SS_{diff}	F	p
979	945.46	-	-	-
978	945.44	0.02	0.02	0.897
977	940.92	4.53	4.83	**0.028**
976	940.88	0.04	0.04	0.844
975	914.41	26.47	28.22	**<.001**

Table S13. Model comparison results for regression models predicting model-based weighting parameter values from winning behavioural model. df_R *= residual degrees of freedom,* SS_R *= residual sum of squares,* SS_{diff} *= difference in residual sum of squares between models,* F *= F-statistic,* p *= p-value.*

	β	β_{SE}	t	p	$CI_{2.5}$	$CI_{97.5}$
Intercept	0.16	0.05	3.35	**0.001**	0.07	0.25
Age	-0.14	0.03	-4.26	**<.001**	-0.20	-0.07
Gender	-0.31	0.07	-4.46	**<.001**	-0.45	-0.17
Motivation	0.08	0.03	2.49	**0.013**	0.02	0.14
Externalising	-0.12	0.04	-3.34	**0.001**	-0.19	-0.05
Internalising	-0.15	0.04	-3.58	**<.001**	-0.23	-0.07
Withdrawal	-0.00	0.04	-0.11	0.911	-0.08	0.07
Inattention	0.20	0.04	5.13	**<.001**	0.13	0.28

Table S14. Coefficients for winning regression model predicting relative fit of model-based and model-free models of movement prediction. β *= regression coefficient,* β_{SE} *= standard error of regression coefficient,* t *= t-statistic,* p *= p-value,* $CI_{2.5}$ *= 95% confidence interval lower bound,* $CI_{97.5}$ *= 95% confidence interval upper bound.*

	β	β_{SE}	t	p	$CI_{2.5}$	$CI_{97.5}$
Intercept	0.12	0.05	2.56	**0.011**	0.03	0.21
Age	-0.17	0.03	-5.04	**<.001**	-0.23	-0.10
Gender	-0.24	0.07	-3.41	**0.001**	-0.38	-0.10
Motivation	0.06	0.03	1.89	0.059	-0.00	0.12
Externalising	-0.12	0.04	-3.52	**<.001**	-0.19	-0.06
Internalising	-0.15	0.04	-3.46	**0.001**	-0.23	-0.06
Withdrawal	0.03	0.04	0.85	0.394	-0.04	0.11
Inattention	0.19	0.04	4.64	**<.001**	0.11	0.26

Table S15. Coefficients for winning regression model predicting model-based weighting parameter values from winning behavioural model. β = regression coefficient, β_{SE} = standard error of regression coefficient, t = t-statistic, p = p-value, $CI_{2.5}$ = 95% confidence interval lower bound, $CI_{97.5}$ = 95% confidence interval upper bound.

Path	β	β_{SE}	p	$CI_{2.5}$	$CI_{97.5}$
relative_BIC ~ X	0.20	0.04	**<.001**	0.13	0.28
Y ~ relative_BIC	0.40	0.03	**<.001**	0.34	0.45
Total	0.19	0.04	**<.001**	0.11	0.27
Direct	0.12	0.04	**0.002**	0.04	0.19
Indirect	0.08	0.02	**<.001**	0.05	0.11

Table S16. Mediation model examining mediation of the inattention > prediction accuracy association by model-basedness (as measured by difference in model fit between model-based and model-free strategies). β = regression coefficient, β_{SE} = standard error of regression coefficient, t = t-statistic, p = p-value, $CI_{2.5}$ = 95% confidence interval lower bound, $CI_{97.5}$ = 95% confidence interval upper bound.

Path	β	β_{SE}	p	$CI_{2.5}$	$CI_{97.5}$
w_values ~ X	0.19	0.04	**<.001**	0.11	0.26
Y ~ w_values	0.39	0.03	**<.001**	0.33	0.45
Total	0.19	0.04	**<.001**	0.11	0.27
Direct	0.12	0.04	**0.001**	0.05	0.20
Indirect	0.07	0.02	**<.001**	0.04	0.10

Table S17. Mediation model examining mediation of the inattention > prediction accuracy association by model-basedness (as measured by difference in model-based weighting parameter values). β = regression coefficient, β_{SE} = standard error of regression coefficient, t = t-statistic, p = p-value, $CI_{2.5}$ = 95% confidence interval lower bound, $CI_{97.5}$ = 95% confidence interval upper bound.

Path	β	β_{SE}	p	$CI_{2.5}$	$CI_{97.5}$
Model fit difference ~ Externalising	-0.12	0.04	**0.001**	-0.19	-0.05
Proportion correct ~ Model fit difference	0.38	0.03	**<.001**	0.33	0.44
Total	-0.07	0.04	**0.038**	-0.14	-0.00
Direct	-0.03	0.03	0.384	-0.09	0.04
Indirect	-0.04	0.01	**0.001**	-0.07	-0.02

Table S18. Mediation model examining mediation of the externalising > prediction accuracy association by model-basedness (as measured by difference in model fit between model-based and model-free strategies). β *= regression coefficient,* β_{SE} *= standard error of regression coefficient,* t *= t-statistic,* p *= p-value,* $CI_{2.5}$ *= 95% confidence interval lower bound,* $CI_{97.5}$ *= 95% confidence interval upper bound.*

Path	β	β_{SE}	p	$CI_{2.5}$	$CI_{97.5}$
Model-based weighting ~ Externalising	-0.12	0.04	**<.001**	-0.19	-0.06
Proportion correct ~ Model-based weighting	0.38	0.03	**<.001**	0.32	0.44
Total	-0.07	0.04	**0.038**	-0.14	-0.00
Direct	-0.03	0.03	0.414	-0.09	0.04
Indirect	-0.05	0.01	**0.001**	-0.08	-0.02

Table S19. Mediation model examining mediation of the externalising > prediction accuracy association by model-basedness (as measured by difference in model-based weightin parameter values). β *= regression coefficient,* β_{SE} *= standard error of regression coefficient,* t *= t-statistic,* p *= p-value,* $CI_{2.5}$ *= 95% confidence interval lower bound,* $CI_{97.5}$ *= 95% confidence interval upper bound.*

Parameter	Measure	β	β_{SE}	t	p	$CI_{2.5}$	$CI_{97.5}$	p_{FDR}
Relative model-based model fit	AQ-10	0.28	0.15	1.89	0.069	-0.03	0.58	0.430
	STICSA-T	-0.04	0.06	-0.60	0.569	-0.16	0.09	0.948
	LSAS	-0.00	0.04	-0.03	0.975	-0.09	0.09	0.975
	PHQ-8	-0.13	0.15	-0.82	0.397	-0.43	0.17	0.795
	GAD-7	-0.29	0.16	-1.86	0.086	-0.61	0.04	0.430
Model-based weighting parameter	AQ-10	0.00	0.00	1.12	0.240	-0.00	0.01	0.793
	STICSA-T	0.00	0.00	0.07	0.944	-0.00	0.00	0.975
	LSAS	-0.00	0.00	-0.04	0.968	-0.00	0.00	0.975
	PHQ-8	-0.00	0.00	-0.27	0.791	-0.00	0.00	0.975
	GAD-7	-0.00	0.00	-1.10	0.317	-0.01	0.00	0.793

Table S20. Coefficients for additional measures predicting different aspects of behaviour, taken from regression models including the same covariates as the primary models. β *= regression coefficient,* β_{SE} *= standard error of regression coefficient,* t *= t-statistic,* p *= p-value,* $CI_{2.5}$ *= 95% confidence interval lower bound,* $CI_{97.5}$ *= 95% confidence interval upper bound., AQ-10 = Autism Spectrum Quotient, STICSA-T = State Trait Inventory of Cognitive and Somatic Anxiety, LSAS = Liebowitz Social Anxiety Scale, PHQ-8 = Patient Health Questionnaire, GAD-7 = Generalized Anxiety Disorder Assessment*

Supplementary references

1 Dam NTV, Gros DF, Earleywine M, Antony MM. Establishing a trait anxiety threshold that signals likelihood of anxiety disorders. *Anxiety, Stress, & Coping* 2013; **26**: 70–86.

2 Kessler RC, Adler L, Ames M, Demler O, Faraone S, Hiripi E *et al*. The World Health Organization adult ADHD self-report scale (ASRS): a short screening scale for use in the general population. *Psychol Med* 2005; **35**: 245–256.

3 Allison C, Auyeung B, Baron-Cohen S. Toward Brief “Red Flags” for Autism Screening: The Short Autism Spectrum Quotient and the Short Quantitative Checklist in 1,000 Cases and 3,000 Controls. *Journal of the American Academy of Child & Adolescent Psychiatry* 2012; **51**: 202-212.e7.

4 Boonstra N, Wunderink L, Sytema S, Wiersma D. Improving detection of first-episode psychosis by mental health-care services using a self-report questionnaire. *Early Intervention in Psychiatry* 2009; **3**: 289–295.
